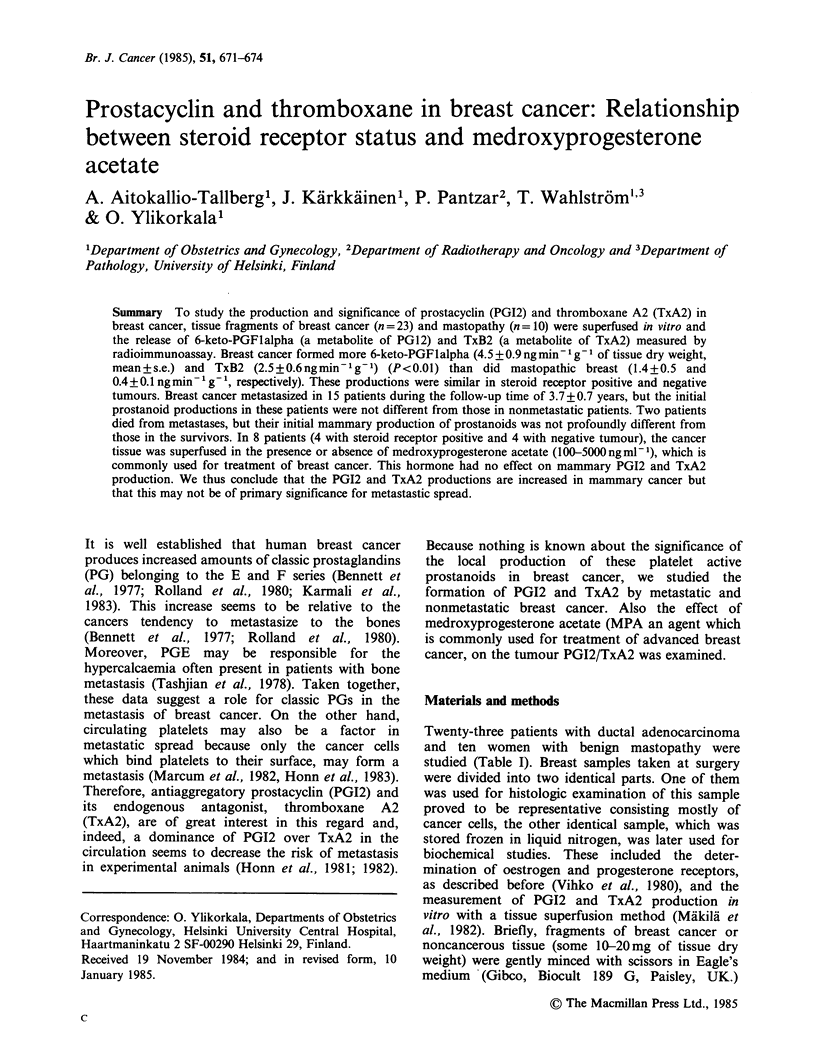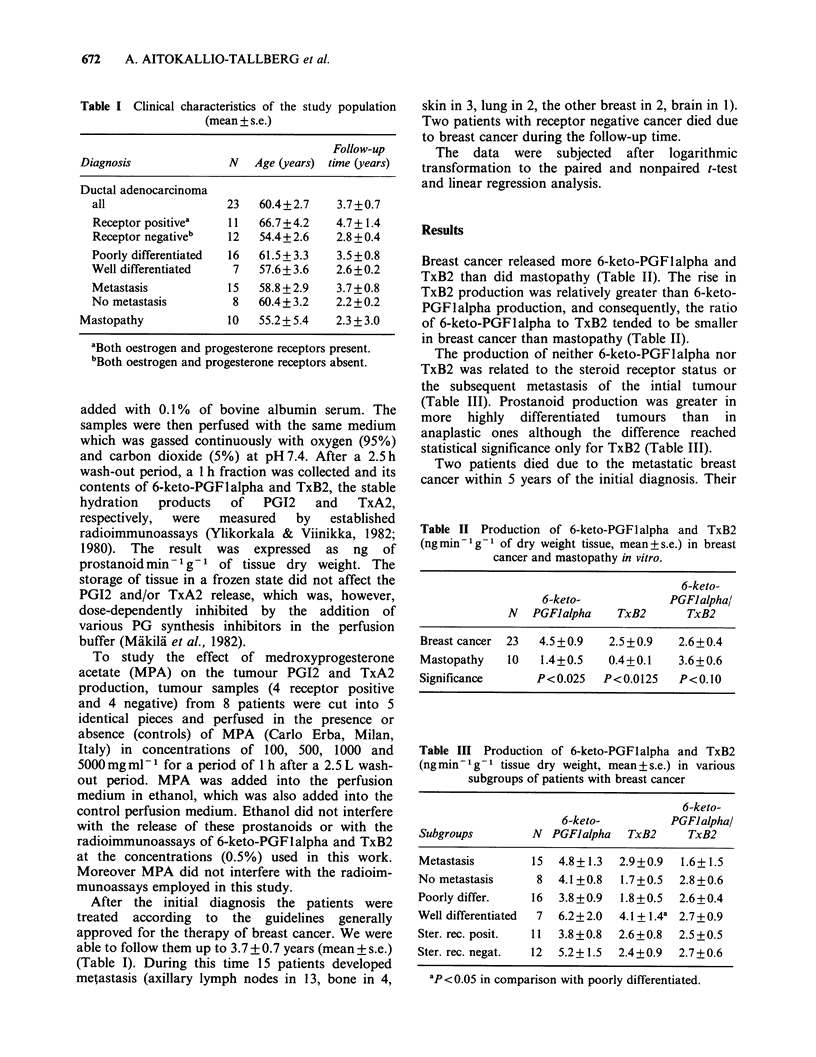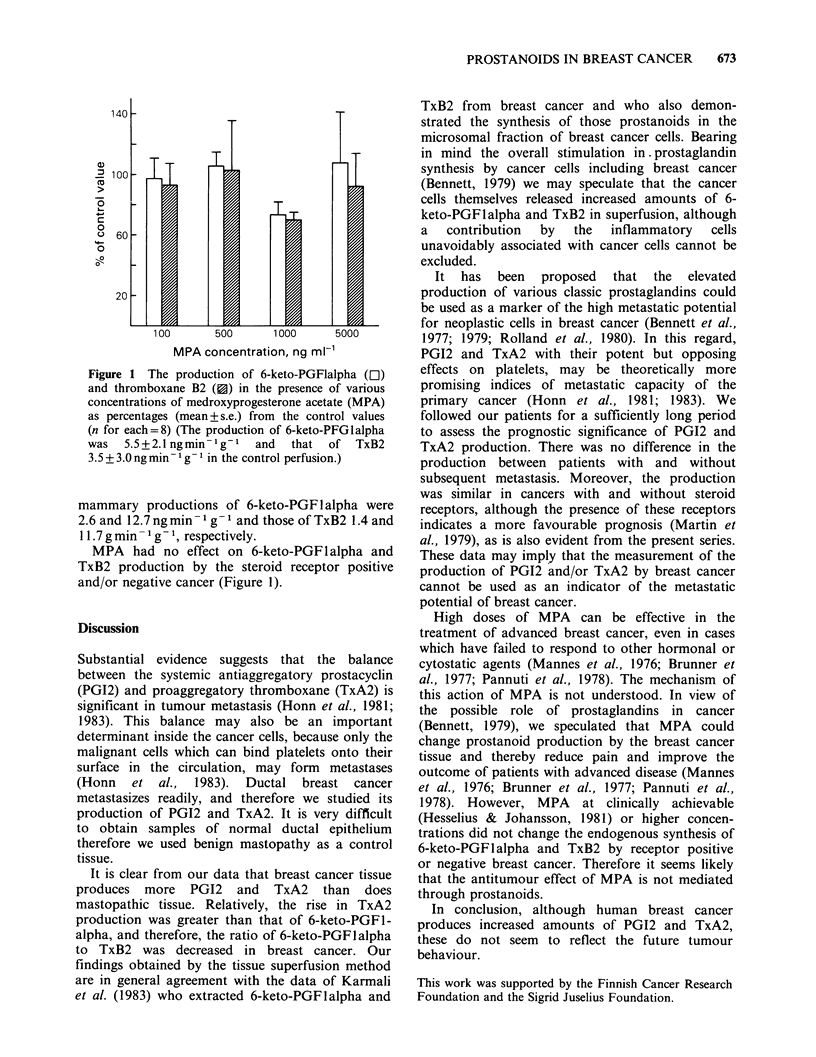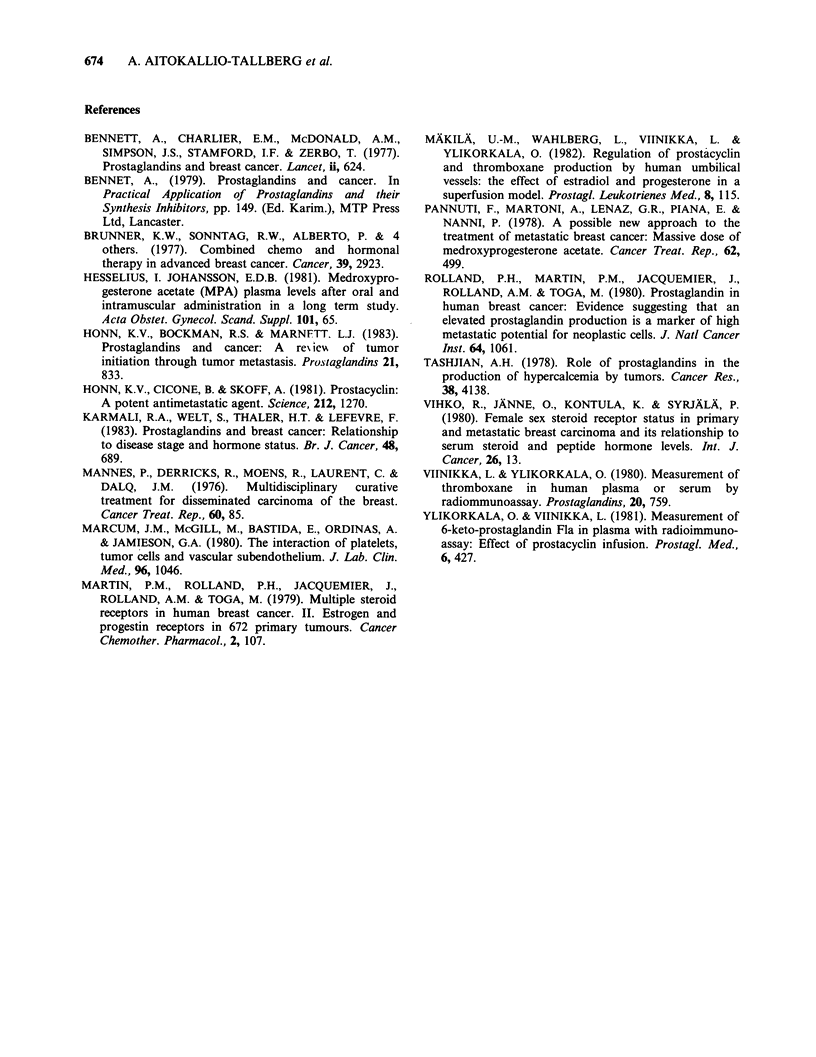# Prostacyclin and thromboxane in breast cancer: relationship between steroid receptor status and medroxyprogesterone acetate.

**DOI:** 10.1038/bjc.1985.101

**Published:** 1985-05

**Authors:** A. Aitokallio-Tallberg, J. Kärkkäinen, P. Pantzar, T. Wahlström, O. Ylikorkala

## Abstract

To study the production and significance of prostacyclin (PGI2) and thromboxane A2 (TxA2) in breast cancer, tissue fragments of breast cancer (n=23) and mastopathy (n=10) were superfused in vitro and the release of 6-keto-PGF1 alpha (a metabolite of PG12) and TxB2 (a metabolite of TxA2) measured by radioimmunoassay. Breast cancer formed more 6-keto-PGF1 alpha (4.5 +/- 0.9 ng min-1 g-1 of tissue dry weight, mean +/- s.e.) and TxB2 (2.5 +/- 0.6 ng min-1 g-1) (P less than 0.01) than did mastopathic breast (1.4 +/- 0.5 and 0.4 +/- 0.1 ng min-1 g-1, respectively). These productions were similar in steroid receptor positive and negative tumours. Breast cancer metastasized in 15 patients during the follow-up time of 3.7 +/- 0.7 years, but the initial prostanoid productions in these patients were not different from those in nonmetastatic patients. Two patients died from metastases, but their initial mammary production of prostanoids was not profoundly different from those in the survivors. In 8 patients (4 with steroid receptor positive and 4 with negative tumour), the cancer tissue was superfused in the presence or absence of medroxyprogesterone acetate (100-5000 ng ml-1), which is commonly used for treatment of breast cancer. This hormone had no effect on mammary PGI2 and TxA2 production. We thus conclude that the PGI2 and TxA2 productions are increased in mammary cancer but that this may not be of primary significance for metastastic spread.


*Br. J. Cancer* (1985), **51**, 671–674

### Prostacyclin and thromboxane in breast cancer: Relationship between steroid receptor status and medroxyprogesterone acetate

A. Aitokallio-Tallberg[1], J. Kärkkäinen[1], P. Pantzar[2], T. Wahlström[1,3] & O. Ylikorkala[1]

[1]*Department of Obstetrics and Gynecology,* [2]*Department of Radiotherapy and Oncology and* [3]*Department of Pathology, University of Helsinki, Finland*

**Summary** To study the production and significance of prostacyclin (PGI2) and thromboxane A2 (TxA2) in breast cancer, tissue fragments of breast cancer ($n=23$) and mastopathy ($n=10$) were superfused *in vitro* and the release of 6-keto-PGF1alpha (a metabolite of PG12) and TxB2 (a metabolite of TxA2) measured by radioimmunoassay. Breast cancer formed more 6-keto-PGF1alpha ($4.5 \pm 0.9\,ng\,min^{-1}\,g^{-1}$ of tissue dry weight, mean $\pm$ s.e.) and TxB2 ($2.5 \pm 0.6\,ng\,min^{-1}\,g^{-1}$) ($P<0.01$) than did mastopathic breast ($1.4 \pm 0.5$ and $0.4 \pm 0.1\,ng\,min^{-1}\,g^{-1}$, respectively). These productions were similar in steroid receptor positive and negative tumours. Breast cancer metastasized in 15 patients during the follow-up time of $3.7 \pm 0.7$ years, but the initial prostanoid productions in these patients were not different from those in nonmetastatic patients. Two patients died from metastases, but their initial mammary production of prostanoids was not profoundly different from those in the survivors. In 8 patients (4 with steroid receptor positive and 4 with negative tumour), the cancer tissue was superfused in the presence or absence of medroxyprogesterone acetate ($100$–$5000\,ng\,ml^{-1}$), which is commonly used for treatment of breast cancer. This hormone had no effect on mammary PGI2 and TxA2 production. We thus conclude that the PGI2 and TxA2 productions are increased in mammary cancer but that this may not be of primary significance for metastastic spread.

It is well established that human breast cancer produces increased amounts of classic prostaglandins (PG) belonging to the E and F series (Bennett *et al.,* 1977; Rolland *et al.,* 1980; Karmali *et al.,* 1983). This increase seems to be relative to the cancers tendency to metastasize to the bones (Bennett *et al.,* 1977; Rolland *et al.,* 1980). Moreover, PGE may be responsible for the hypercalcaemia often present in patients with bone metastasis (Tashjian *et al.,* 1978). Taken together, these data suggest a role for classic PGs in the metastasis of breast cancer. On the other hand, circulating platelets may also be a factor in metastatic spread because only the cancer cells which bind platelets to their surface, may form a metastasis (Marcum *et al.,* 1982, Honn *et al.,* 1983). Therefore, antiaggregatory prostacyclin (PGI2) and its endogenous antagonist, thromboxane A2 (TxA2), are of great interest in this regard and, indeed, a dominance of PGI2 over TxA2 in the circulation seems to decrease the risk of metastasis in experimental animals (Honn *et al.,* 1981; 1982).

Correspondence: O. Ylikorkala, Departments of Obstetrics and Gynecology, Helsinki University Central Hospital, Haartmaninkatu 2 SF-00290 Helsinki 29, Finland.

Received 19 November 1984; and in revised form, 10 January 1985.

Because nothing is known about the significance of the local production of these platelet active prostanoids in breast cancer, we studied the formation of PGI2 and TxA2 by metastatic and nonmetastatic breast cancer. Also the effect of medroxyprogesterone acetate (MPA an agent which is commonly used for treatment of advanced breast cancer, on the tumour PGI2/TxA2 was examined.

### Materials and methods

Twenty-three patients with ductal adenocarcinoma and ten women with benign mastopathy were studied (Table I). Breast samples taken at surgery were divided into two identical parts. One of them was used for histologic examination of this sample proved to be representative consisting mostly of cancer cells, the other identical sample, which was stored frozen in liquid nitrogen, was later used for biochemical studies. These included the determination of oestrogen and progesterone receptors, as described before (Vihko *et al.,* 1980), and the measurement of PGI2 and TxA2 production *in vitro* with a tissue superfusion method (Mäkilä *et al.,* 1982). Briefly, fragments of breast cancer or noncancerous tissue (some 10–20 mg of tissue dry weight) were gently minced with scissors in Eagle's medium (Gibco, Biocult 189 G, Paisley, UK.)

© The Macmillan Press Ltd., 1985

**Table I** Clinical characteristics of the study population (mean ± s.e.)

| *Diagnosis* | *N* | *Age (years)* | *Follow-up time (years)* |
|---|---|---|---|
| Ductal adenocarcinoma | | | |
| all | 23 | 60.4±2.7 | 3.7±0.7 |
| Receptor positive[a] | 11 | 66.7±4.2 | 4.7±1.4 |
| Receptor negative[b] | 12 | 54.4±2.6 | 2.8±0.4 |
| Poorly differentiated | 16 | 61.5±3.3 | 3.5±0.8 |
| Well differentiated | 7 | 57.6±3.6 | 2.6±0.2 |
| Metastasis | 15 | 58.8±2.9 | 3.7±0.8 |
| No metastasis | 8 | 60.4±3.2 | 2.2±0.2 |
| Mastopathy | 10 | 55.2±5.4 | 2.3±3.0 |

[a]Both oestrogen and progesterone receptors present.
[b]Both oestrogen and progesterone receptors absent.

added with 0.1% of bovine albumin serum. The samples were then perfused with the same medium which was gassed continuously with oxygen (95%) and carbon dioxide (5%) at pH 7.4. After a 2.5 h wash-out period, a 1 h fraction was collected and its contents of 6-keto-PGF1alpha and TxB2, the stable hydration products of PGI2 and TxA2, respectively, were measured by established radioimmunoassays (Ylikorkala & Viinikka, 1982; 1980). The result was expressed as ng of prostanoid $min^{-1} g^{-1}$ of tissue dry weight. The storage of tissue in a frozen state did not affect the PGI2 and/or TxA2 release, which was, however, dose-dependently inhibited by the addition of various PG synthesis inhibitors in the perfusion buffer (Mäkilä *et al.*, 1982).

To study the effect of medroxyprogesterone acetate (MPA) on the tumour PGI2 and TxA2 production, tumour samples (4 receptor positive and 4 negative) from 8 patients were cut into 5 identical pieces and perfused in the presence or absence (controls) of MPA (Carlo Erba, Milan, Italy) in concentrations of 100, 500, 1000 and 5000 $mg\,ml^{-1}$ for a period of 1 h after a 2.5 L wash-out period. MPA was added into the perfusion medium in ethanol, which was also added into the control perfusion medium. Ethanol did not interfere with the release of these prostanoids or with the radioimmunoassays of 6-keto-PGF1alpha and TxB2 at the concentrations (0.5%) used in this work. Moreover MPA did not interfere with the radioimmunoassays employed in this study.

After the initial diagnosis the patients were treated according to the guidelines generally approved for the therapy of breast cancer. We were able to follow them up to 3.7±0.7 years (mean±s.e.) (Table I). During this time 15 patients developed metastasis (axillary lymph nodes in 13, bone in 4, skin in 3, lung in 2, the other breast in 2, brain in 1). Two patients with receptor negative cancer died due to breast cancer during the follow-up time.

The data were subjected after logarithmic transformation to the paired and nonpaired *t*-test and linear regression analysis.

### Results

Breast cancer released more 6-keto-PGF1alpha and TxB2 than did mastopathy (Table II). The rise in TxB2 production was relatively greater than 6-keto-PGF1alpha production, and consequently, the ratio of 6-keto-PGF1alpha to TxB2 tended to be smaller in breast cancer than mastopathy (Table II).

The production of neither 6-keto-PGF1alpha nor TxB2 was related to the steroid receptor status or the subsequent metastasis of the intial tumour (Table III). Prostanoid production was greater in more highly differentiated tumours than in anaplastic ones although the difference reached statistical significance only for TxB2 (Table III).

Two patients died due to the metastatic breast cancer within 5 years of the initial diagnosis. Their

**Table II** Production of 6-keto-PGF1alpha and TxB2 (ng $min^{-1} g^{-1}$ of dry weight tissue, mean±s.e.) in breast cancer and mastopathy *in vitro*.

| | *N* | *6-keto-PGF1alpha* | *TxB2* | *6-keto-PGF1alpha/TxB2* |
|---|---|---|---|---|
| Breast cancer | 23 | 4.5±0.9 | 2.5±0.9 | 2.6±0.4 |
| Mastopathy | 10 | 1.4±0.5 | 0.4±0.1 | 3.6±0.6 |
| Significance | | $P<0.025$ | $P<0.0125$ | $P<0.10$ |

**Table III** Production of 6-keto-PGF1alpha and TxB2 (ng $min^{-1} g^{-1}$ tissue dry weight, mean±s.e.) in various subgroups of patients with breast cancer

| *Subgroups* | *N* | *6-keto-PGF1alpha* | *TxB2* | *6-keto-PGF1alpha/TxB2* |
|---|---|---|---|---|
| Metastasis | 15 | 4.8±1.3 | 2.9±0.9 | 1.6±1.5 |
| No metastasis | 8 | 4.1±0.8 | 1.7±0.5 | 2.8±0.6 |
| Poorly differ. | 16 | 3.8±0.9 | 1.8±0.5 | 2.6±0.4 |
| Well differentiated | 7 | 6.2±2.0 | 4.1±1.4[a] | 2.7±0.9 |
| Ster. rec. posit. | 11 | 3.8±0.8 | 2.6±0.8 | 2.5±0.5 |
| Ster. rec. negat. | 12 | 5.2±1.5 | 2.4±0.9 | 2.7±0.6 |

[a]$P<0.05$ in comparison with poorly differentiated.

**Figure 1** The production of 6-keto-PGFlalpha (□) and thromboxane B2 (▨) in the presence of various concentrations of medroxyprogesterone acetate (MPA) as percentages (mean ± s.e.) from the control values (*n* for each = 8) (The production of 6-keto-PFG1alpha was $5.5 \pm 2.1\ \mathrm{ng\,min^{-1}\,g^{-1}}$ and that of TxB2 $3.5 \pm 3.0\ \mathrm{ng\,min^{-1}\,g^{-1}}$ in the control perfusion.)

mammary productions of 6-keto-PGF1alpha were 2.6 and $12.7\ \mathrm{ng\,min^{-1}\,g^{-1}}$ and those of TxB2 1.4 and $11.7\ \mathrm{g\,min^{-1}\,g^{-1}}$, respectively.

MPA had no effect on 6-keto-PGF1alpha and TxB2 production by the steroid receptor positive and/or negative cancer (Figure 1).

### Discussion

Substantial evidence suggests that the balance between the systemic antiaggregatory prostacyclin (PGI2) and proaggregatory thromboxane (TxA2) is significant in tumour metastasis (Honn *et al.*, 1981; 1983). This balance may also be an important determinant inside the cancer cells, because only the malignant cells which can bind platelets onto their surface in the circulation, may form metastases (Honn *et al.*, 1983). Ductal breast cancer metastasizes readily, and therefore we studied its production of PGI2 and TxA2. It is very difficult to obtain samples of normal ductal epithelium therefore we used benign mastopathy as a control tissue.

It is clear from our data that breast cancer tissue produces more PGI2 and TxA2 than does mastopathic tissue. Relatively, the rise in TxA2 production was greater than that of 6-keto-PGF1-alpha, and therefore, the ratio of 6-keto-PGF1alpha to TxB2 was decreased in breast cancer. Our findings obtained by the tissue superfusion method are in general agreement with the data of Karmali *et al.* (1983) who extracted 6-keto-PGF1alpha and TxB2 from breast cancer and who also demonstrated the synthesis of those prostanoids in the microsomal fraction of breast cancer cells. Bearing in mind the overall stimulation in prostaglandin synthesis by cancer cells including breast cancer (Bennett, 1979) we may speculate that the cancer cells themselves released increased amounts of 6-keto-PGF1alpha and TxB2 in superfusion, although a contribution by the inflammatory cells unavoidably associated with cancer cells cannot be excluded.

It has been proposed that the elevated production of various classic prostaglandins could be used as a marker of the high metastatic potential for neoplastic cells in breast cancer (Bennett *et al.*, 1977; 1979; Rolland *et al.*, 1980). In this regard, PGI2 and TxA2 with their potent but opposing effects on platelets, may be theoretically more promising indices of metastatic capacity of the primary cancer (Honn *et al.*, 1981; 1983). We followed our patients for a sufficiently long period to assess the prognostic significance of PGI2 and TxA2 production. There was no difference in the production between patients with and without subsequent metastasis. Moreover, the production was similar in cancers with and without steroid receptors, although the presence of these receptors indicates a more favourable prognosis (Martin *et al.*, 1979), as is also evident from the present series. These data may imply that the measurement of the production of PGI2 and/or TxA2 by breast cancer cannot be used as an indicator of the metastatic potential of breast cancer.

High doses of MPA can be effective in the treatment of advanced breast cancer, even in cases which have failed to respond to other hormonal or cytostatic agents (Mannes *et al.*, 1976; Brunner *et al.*, 1977; Pannuti *et al.*, 1978). The mechanism of this action of MPA is not understood. In view of the possible role of prostaglandins in cancer (Bennett, 1979), we speculated that MPA could change prostanoid production by the breast cancer tissue and thereby reduce pain and improve the outcome of patients with advanced disease (Mannes *et al.*, 1976; Brunner *et al.*, 1977; Pannuti *et al.*, 1978). However, MPA at clinically achievable (Hesselius & Johansson, 1981) or higher concentrations did not change the endogenous synthesis of 6-keto-PGF1alpha and TxB2 by receptor positive or negative breast cancer. Therefore it seems likely that the antitumour effect of MPA is not mediated through prostanoids.

In conclusion, although human breast cancer produces increased amounts of PGI2 and TxA2, these do not seem to reflect the future tumour behaviour.

This work was supported by the Finnish Cancer Research Foundation and the Sigrid Juselius Foundation.

### References

BENNETT, A., CHARLIER, E.M., McDONALD, A.M., SIMPSON, J.S., STAMFORD, I.F. & ZERBO, T. (1977). Prostaglandins and breast cancer. *Lancet*, **ii**, 624.

BENNET, A., (1979). Prostaglandins and cancer. In *Practical Application of Prostaglandins and their Synthesis Inhibitors*, pp. 149. (Ed. Karim.), MTP Press Ltd, Lancaster.

BRUNNER, K.W., SONNTAG, R.W., ALBERTO, P. & 4 others. (1977). Combined chemo and hormonal therapy in advanced breast cancer. *Cancer*, **39**, 2923.

HESSELIUS, I. JOHANSSON, E.D.B. (1981). Medroxyprogesterone acetate (MPA) plasma levels after oral and intramuscular administration in a long term study. *Acta Obstet. Gynecol. Scand. Suppl.* **101**, 65.

HONN, K.V., BOCKMAN, R.S. & MARNETT, L.J. (1983). Prostaglandins and cancer: A review of tumor initiation through tumor metastasis. *Prostaglandins* **21**, 833.

HONN, K.V., CICONE, B. & SKOFF, A. (1981). Prostacyclin: A potent antimetastatic agent. *Science*, **212**, 1270.

KARMALI, R.A., WELT, S., THALER, H.T. & LEFEVRE, F. (1983). Prostaglandins and breast cancer: Relationship to disease stage and hormone status. *Br. J. Cancer*, **48**, 689.

MANNES, P., DERRICKS, R., MOENS, R., LAURENT, C. & DALQ, J.M. (1976). Multidisciplinary curative treatment for disseminated carcinoma of the breast. *Cancer Treat. Rep.*, **60**, 85.

MARCUM, J.M., McGILL, M., BASTIDA, E., ORDINAS, A. & JAMIESON, G.A. (1980). The interaction of platelets, tumor cells and vascular subendothelium. *J. Lab. Clin. Med.*, **96**, 1046.

MARTIN, P.M., ROLLAND, P.H., JACQUEMIER, J., ROLLAND, A.M. & TOGA, M. (1979). Multiple steroid receptors in human breast cancer. II. Estrogen and progestin receptors in 672 primary tumours. *Cancer Chemother. Pharmacol.*, **2**, 107.

MÄKILÄ, U.-M., WAHLBERG, L., VIINIKKA, L. & YLIKORKALA, O. (1982). Regulation of prostacyclin and thromboxane production by human umbilical vessels: the effect of estradiol and progesterone in a superfusion model. *Prostagl. Leukotrienes Med.*, **8**, 115.

PANNUTI, F., MARTONI, A., LENAZ, G.R., PIANA, E. & NANNI, P. (1978). A possible new approach to the treatment of metastatic breast cancer: Massive dose of medroxyprogesterone acetate. *Cancer Treat. Rep.*, **62**, 499.

ROLLAND, P.H., MARTIN, P.M., JACQUEMIER, J., ROLLAND, A.M. & TOGA, M. (1980). Prostaglandin in human breast cancer: Evidence suggesting that an elevated prostaglandin production is a marker of high metastatic potential for neoplastic cells. *J. Natl Cancer Inst.* **64**, 1061.

TASHJIAN, A.H. (1978). Role of prostaglandins in the production of hypercalcemia by tumors. *Cancer Res.*, **38**, 4138.

VIHKO, R., JÄNNE, O., KONTULA, K. & SYRJÄLÄ, P. (1980). Female sex steroid receptor status in primary and metastatic breast carcinoma and its relationship to serum steroid and peptide hormone levels. *Int. J. Cancer*, **26**, 13.

VIINIKKA, L. & YLIKORKALA, O. (1980). Measurement of thromboxane in human plasma or serum by radiommunoassay. *Prostaglandins*, **20**, 759.

YLIKORKALA, O. & VIINIKKA, L. (1981). Measurement of 6-keto-prostaglandin Fla in plasma with radioimmunoassay: Effect of prostacyclin infusion. *Prostagl. Med.*, **6**, 427.